# A Th2-score in the tumor microenvironment as a predictive biomarker of response to Bacillus Calmette Guérin in patients with non-muscle invasive bladder carcinoma: A retrospective study

**DOI:** 10.32604/or.2023.028163

**Published:** 2023-04-10

**Authors:** GUSTAVO MARTÍN VILLOLDO, MARÍA TERESA POMBO, MARIANA ARIS, JOAQUÍN CHEMI, PABLO MANDÓ, SUPRIYA NAGARAJU, JUAN CAMEAN, ADRIÁN BURIONI, DEBORAH EGEA, MORA AMAT, JOSÉ LEÓN MELLADO, JOSÉ MORDOH, ALBERTO VILLARONGA, MARÍA MARCELA BARRIO

**Affiliations:** 1 Urology Department, Instituto Alexander Fleming, Ciudad Autónoma de Buenos Aires, 1426, Argentina; 2 Immunohistochemistry Department, Instituto Alexander Fleming, Ciudad Autónoma de Buenos Aires, 1426, Argentina; 3 Centro de Investigaciones Oncológicas, Fundación Cáncer FUCA, Ciudad Autónoma de Buenos Aires, 1426, Argentina; 4 Urology Department, MD Anderson Cancer Center, Houston, TX, 77030, USA; 5 Pathology Department, Instituto Alexander Fleming, Ciudad Autónoma de Buenos Aires, 1426, Argentina

**Keywords:** Non-muscle invasive bladder cancer, BCG predictive biomarkers, Lymphocyte polarization

## Abstract

Intravesical Bacillus Calmette Guerin (BCG) is the gold standard therapy for intermediate/high-risk non-muscle invasive bladder cancer (NMIBC). However, the response rate is ~60%, and 50% of non-responders will progress to muscle-invasive disease. BCG induces massive local infiltration of inflammatory cells (Th1) and ultimately cytotoxic tumor elimination. We searched for predictive biomarker of BCG response by analyzing tumor-infiltrating lymphocyte (TIL) polarization in the tumor microenvironment (TME) in pre-treatment biopsies. Pre-treatment biopsies from patients with NMIBC who received adequate intravesical instillation of BCG (n = 32) were evaluated retrospectively by immunohistochemistry. TME polarization was assessed by quantifying the T-Bet+ (Th1) and GATA-3+ (Th2) lymphocyte ratio (G/T), and the density and degranulation of EPX+ eosinophils. In addition, PD-1/PD-L1 staining was quantified. The results correlated with BCG response. In most non-responders, Th1/Th2 markers were compared in pre-and post-BCG biopsies. ORR was 65.6% in the study population. BCG responders had a higher G/T ratio and a greater number of degranulated EPX+ cells. Variables combined into a Th2-score showed a significant association with higher scores in responders (*p* = 0.027). A Th2-score cut-off value >48.1 allowed discrimination of responders with 91% sensitivity but lower specificity. Relapse-free survival was significantly associated with the Th2-score (*p* = 0.007). In post-BCG biopsies from recurring patients, TILs increased Th2-polarization, probably reflecting BCG failure to induce a pro-inflammatory status and, thus, a lack of response. PD-L1/PD-1 expression was not associated with the response to BCG. Our results support the hypothesis that a pre-existing Th2-polarized TME predicts a better response to BCG, assuming a reversion to Th1 polarization and antitumor activity.

## Introduction

Non–muscle invasive bladder cancer (NMIBC) represents 70% of all initial bladder cancer diagnoses [1,2]. Some high-risk patients (high-grade [HG] pT1 or carcinoma *in situ* [C*is*]) [3] respond to bladder-conservative treatments and behave in a non-lethal fashion, while others are aggressive and tend to progress to muscle invasion and even metastatic disease. pT1 tumors are mostly HG and progress in >50% of cases; deaths occur in 25% of patients during the first five years [3]. C*is* is mostly concomitant, with increasing recurrence rates from 43% to 73% [4]. Even if C*is* is primary or concomitant, 50% of patients progress to muscle invasion and 20% will ultimately die of metastases if a radical cystectomy is not performed [5]. Intravesical administration of live-attenuated Bacillus Calmette-Guérin (BCG) is the primary adjuvant treatment for high-risk NMIBC patients [6]. Nonetheless, the response rate (RR) to BCG is about 60%, with a 5-year recurrence rate of 30%–40% [7]. Moreover, for those patients presenting either pT1 or C*is*, or both tumors, who do not respond to BCG, the risk of progression to invasive disease may reach 50% [8]. Thus, there is still a strong need to find appropriate predictive biomarkers of response to BCG that may help the selection of patients with a low chance of response, before or early after treatment starts, to offer them alternative therapies. Although the exact mechanism of BCG antitumor activity is not fully understood, it is recognized that BCG acts as a localized immunomodulatory agent promoting Th1 immune polarization, and its efficacy must be related to the immune response elicited in the bladder [9].

GATA binding protein 3 (GATA-3) is a transcription factor that is critical for T cell development and Th2 differentiation [10]. T-box transcription factor 21 (T-Bet) is mostly expressed in CD4+ Th1 cells, and its expression is induced by a combination of T cell receptors and IFN-γ signaling [11]. In addition, T-Bet promotes Th1 polarization by preventing GATA-3-mediated Th2 cell development [12].

In the past, eosinophils were inaccurately considered destructive end-stage effector cells (helminth infections and allergy/asthma) due to the release of toxic cationic granule proteins and the production of reactive oxygen species. More recently, eosinophils have been shown to play a fundamental role in remodeling/repair activities, and more importantly, to modulate local immunity [13].

An emerging concept relates to the prognostic and/or predictive value of Th1/Th2 polarization of tumor-infiltrating cells (TILs) before treatment, which may affect the response to BCG immunotherapy. Interestingly, in patients with C*is* treated with BCG induction alone [14], those who responded to BCG showed an increased density of intratumor Th2 cells and a higher Th2/Th1 ratio prior to treatment, suggesting that BCG would be effective only when the tumor microenvironment (TME) has shifted from Th2 to Th1.

Programmed death ligand 1 (PD-L1) expressed by tumor and immune cells in the TME suppresses antitumor immune responses and promotes tumor progression [15]. However, PD-L1+ TILs have been correlated with improved overall survival in patients with metastatic urothelial cancer [16]. In a recent study, no association of BCG response with peritumoral PD-L1 expression in pretreatment biopsies was reported [17]. On the contrary, high PD-L1 expression in BCG-induced granulomas has been associated with resistance to therapy in urothelial carcinomas [18]. These data suggest that high numbers of PD-L1+ cells in the bladder could suppress the effectiveness of BCG, a hypothesis also supported by the recent demonstration that combined BCG and anti-PD-L1 treatment increased antitumor immunity in an immunocompetent orthotopic rat bladder cancer model [19]. Thus, the PD-1/PD-L1 axis could be involved in the antitumor immune response of NMIBC.

The aim of our study was to identify potential biomarkers to predict the response to BCG in a high-risk population of NMIBC patients, including those with HG pTa, pT1, and C*is* tumors, focusing on Th1/Th2 lymphocyte polarization in the TME. We also explored a possible association of PD-1/PD-L1 expression in NMIBC biopsies with BCG response.

## Materials and Methods

### Patients and BCG immunotherapy

This retrospective review of the bladder cancer registry and biopsies from the Urology Department of Instituto Alexander Fleming (Buenos Aires, Argentina) included patients who were diagnosed between 2007 and 2019. Archive tumor samples were analyzed in this study, and all patients provided written informed consent for the use of biopsies for research purposes at the time of surgery. Patients with NMIBC were stratified according to the EAU risk stratification guidelines [20]. We analyzed the initial bladder tumor biopsy obtained via complete transurethral resection (TURB) and post-treatment biopsies when available. Re-TURB was performed when pT1 NMIBC was detected during the first TURB. The patients did not receive any previous treatment before surgery. Adjuvant treatment started 3–4 weeks after TURB and consisted of a standard 6-dose induction course plus a 3-dose maintenance course at 3, 6, 12, 18, 24, 30, and 36 months of BCG (120 mg of Danish strain 1331 SSI) [21]. The patients did not receive any other intravesical therapy during or after TURB. Follow-up cystoscopy, cytology, and TC scans were performed according to the guidelines [20]. Patients were classified as non-responders (NR) if they had a positive biopsy result for bladder carcinoma after BCG induction plus one maintenance course (adequate BCG). BCG responders (R) were defined as those without any recurrence or evidence of disease based on follow-up cystoscopy, urinary cytology, or re-biopsy. Patients with a history of immunosuppressive drug treatment or other possible confounding factors were excluded.

### Immunohistochemical staining

The biopsies were fixed in formalin and paraffin-embedded. Paraffin blocks containing sufficient material were selected for immunohistochemical (IHC) staining with the following anti-human monoclonal Abs: eosinophil peroxidase EPX-mAb (clone MM 25-82.2, kindly provided by Mayo Clinic, Scottsdale, USA), GATA-3 (clone L50-823, Cell Marque, Rocklin, USA), T-Bet (clone EPR9302 RabMab, Abcam, Cambridge, UK), and desmin to confirm muscle indemnity (clone D33, Dako, Glostrup, Denmark). The sections were stained with anti-human PD-L1 (clone SP263, Ventana, Bend USA) and anti-human PD-1 (clone NAT105, Abcam, Cambridge, UK). All markers, except for PD-L1, were determined using standardized automated protocols for LEICA BOND MAX II. PD-L1 expression was determined using the Benchmark ULTRA (Roche, Basel, Switzerland). Sections were examined by optical microscopy (Olympus BX40 microscope, DP2-BSW software), and digitalized images were analyzed using ImageJ software (NIH).

### Quantitation and scoring

For each immune population, IHC staining was quantified as the average of positive cells in eight high-magnification fields (400×) covering a 1.2 mm^2^ area. EPX+ eosinophils were quantified at the maximum focus of eosinophilic infiltration (Eo count). Also, EPX-mAb staining was used to identify the maximum level of eosinophilic degranulation (Eodgn). A numerical value was given to each level of eosinophil degranulation based on the normal distribution of the data as follows: 0 = no degranulation; 1 = 1a, small sharp granules or 1b, granules with fuzzy edges; and 2 = multiple granules of imprecise edges far from the cell body. GATA-3+ and T-Bet+ lymphocytes were quantified at the maximum focus of mononuclear cell infiltration. The ratio of Th2 polarized (GATA-3+)/Th1 polarized (T-Bet+) lymphocytes (G/T) was calculated. A Th2-score was defined as:


Th2-Score=G/T+(Eo counts∗Eodgn)

PD-L1 expression was quantitated by the combined positive score (CPS), which is the number of PD-L1+ cells (tumor cells, lymphocytes, macrophages) divided by the total number of tumor cells, ×100). Percentages >1% were considered positive for PD-L1. To control for inter-observer variability, all IHC counts were performed in duplicate by two double-blinded researchers. Researcher 1 counts were used because the difference between counts was <10%.

### Statistical analysis

Independent categorical variables were described as percentages and compared using Fisher’s exact test or the chi-square test. Comparisons between R and NR patients were performed using the nonparametric Mann–Whitney U test. Spearman’s correlation coefficient was used to identify associations between continuous variables. GraphPad Prism v10 (GraphPad Software Inc., La Jolla, CA, USA) was used for all statistical analyses. The predictive power of biomarker levels on the BCG response was evaluated by plotting receiver operating characteristic (ROC) curves and calculating the area under the curve (AUC). Recurrence-free survival (RFS) was compared using the Kaplan–Meier method, stratifying patients by the Th2-score and other variables with the log-rank test using R. The *p* values were two-sided, and *p* < 0.05 was considered statistically significant.

## Results

### Response to BCG treatment

In total, 32 patients were included in this retrospective study (Table 1). The mean age at diagnosis was 64.1 years. The histopathological evaluation confirmed primary high-risk NMIBC according to the EAU guidelines. Six patients showed LG, while 26 (81.2%) had HG cancer, according to the WHO 2004/2016 classification. Most patients had pT1 (24/32) and pTa tumors (6/32). Two patients had only C*is*, and two patients had pT1HG + C*is* and pTa LG + C*is*. One patient had concurrent prostate cancer, which was treated with radical prostatectomy at the time of NMIBC diagnosis.

**Table 1:** Clinicopathological parameters associated with BCG immunotherapy response

	BCG responders n = 21	BCG non-responders n = 11	*p* value
Gender, n (%) Male Female	14 (71.4) 7 (54.5)	6 (28.6) 5 (45.5)	0.70*
Age, median (range)	66 (31–82)	61 (52–71)	0.68*
≤65	8 (61.5)	5 (38.5)	
>65	3 (68.4)	6 (31.6)	
Stage, n (%)			0.14**
Ta	2 (33.3)	4 (44.7)	
pT1	18 (75)	6 (25)	
Cis***	3 (75)	1 (25)	
Grade, n (%)			0.15*
Low	2 (33.3)	4 (66.7)	
High	19 (73.1)	7 (26.9)	

Note: *Fisher’s exact test; **Chi-Square test; ***Including both isolated and concomitant C*is* in 2 patients each.

The median follow-up period for the patients without recurrence was 37 months. The pathological finding at post-BCG control was at pT0 in 24 patients. Overall response rate (ORR) to BCG treatment was 65.6%, as 21/32 patients remained recurrence-free for at least 20 months. BCG NR had a median time to recurrence of 15 months; however, 7/11 (63.3%) recurred before 12 months. ORR was not significantly associated with sex, age, tumor stage, or histological grade in the study population. Three of the 11 patients who experienced BCG failure progressed to muscle-invasive disease.

### Tumor-infiltrating lymphocytes profile

**Figure 1:**
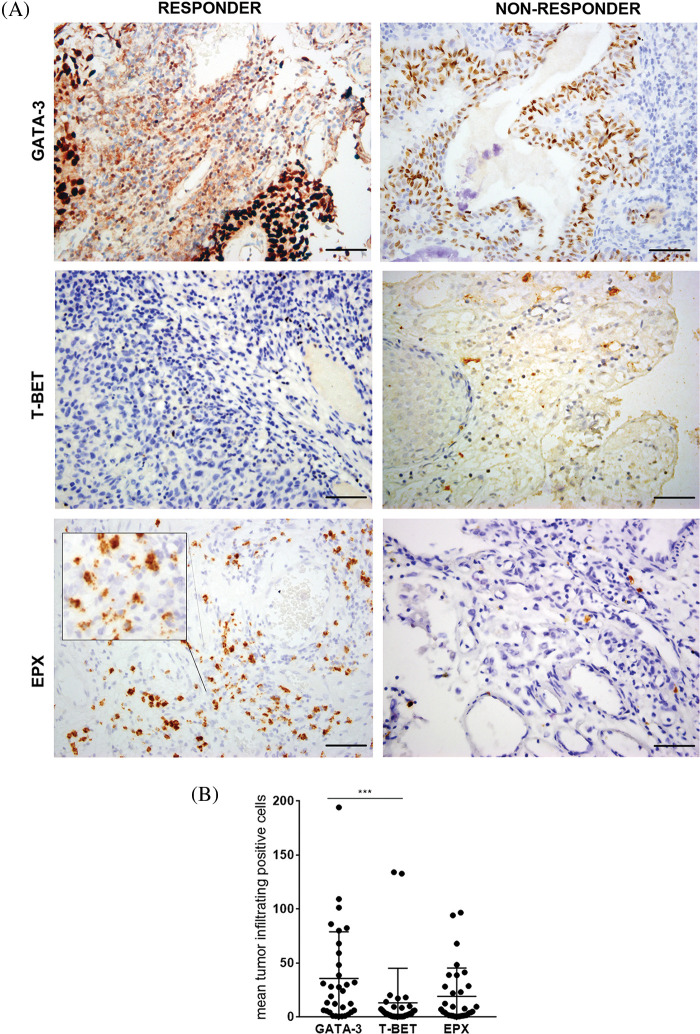
Expression of GATA-3, T-Bet, and EPX in all NMIBC pre-BCG biopsies. (A) Representative pictures of immunohistochemistry staining for GATA-3, T-Bet, and EPX in pre-BCG NMIBC biopsies of R and NR patients. Original magnification 400×. Scale bar = 50 μm. In the case of EPX staining, an inset shows Eodgn in detail. (B) Quantitation of GATA-3+, T-Bet+ TILs, and EPX+ eosinophils EPX in all pre-BCG NMIBC biopsies analyzed. ***Highly significant, *p* = 0.0005, Mann–Whitney test.

The expression of T-Bet and GATA-3 was detected in the TILs of all pre-BCG treatment biopsies (Fig. 1A). Additionally, eosinophils infiltrating the tumor area were detected in 31/32 biopsies (median Eo count 5.8; range 0–96.5). It was possible to evaluate the extent of Eo degranulation (Eodgn), as a readout of its activation in the TME, because EPX+ granules were evident. A significantly higher infiltration of Th2 cells than Th1 cells was identified, with a median (range) number of total GATA-3+ and T-Bet+ cells of 21.5 (0.5–194) and 2.9 (0.125–134), respectively (*p* < 0.0005) (Fig. 1B and Suppl. Table S1).

No significant correlation was found between the total number of GATA-3+ and T-Bet+ cells, GATA-3+ and Eo counts, or Eodgn or T-Bet+ and EPX+ cells or Eodgn. Only Eo counts correlated with Eodgn, suggesting that when eosinophils were abundant in the TME, most were activated (Suppl. Table S2).

**FIGURE S1:**
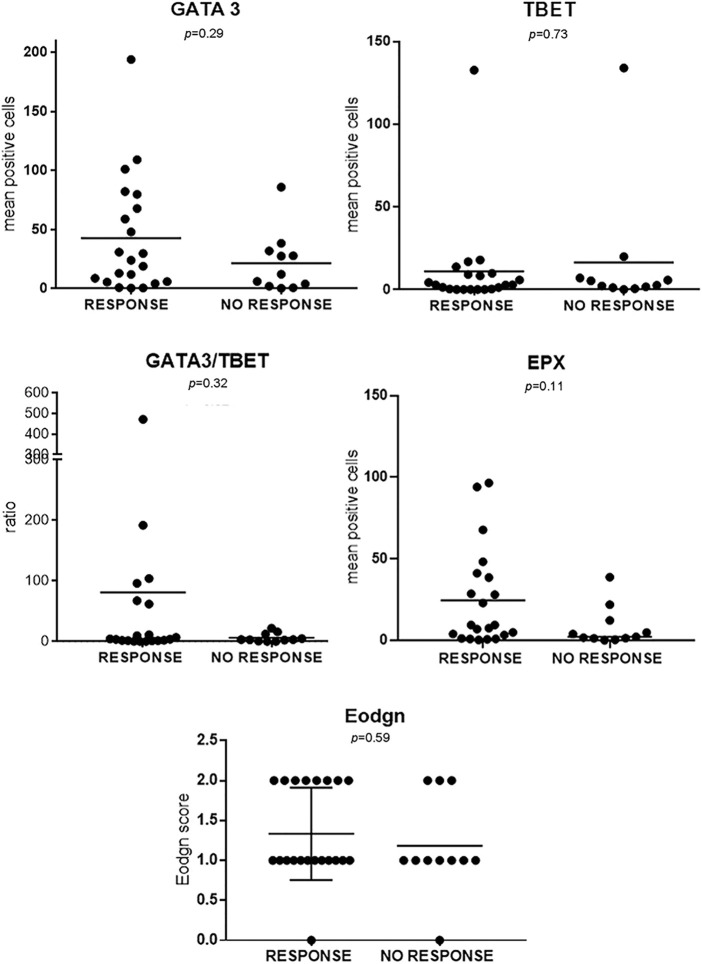
Each variable quantitated by IHQ was plotted for BCG-R and BCG-NR patients (n = 32).

TIL expression of GATA-3, T-Bet, and EPX (Eo counts or Eodgn) was compared in biopsies from BCG-R and BCG-NR patients. The BCG response was associated with a higher number of GATA-3+ and EPX+ cells, a higher GATA-3/T-Bet ratio (G/T), and a lower number of T-Bet+ cells, although these differences were not statistically significant (Fig. 1, Suppl. Fig. S1).

### Th2-score predicts BCG response

**Figure 2:**
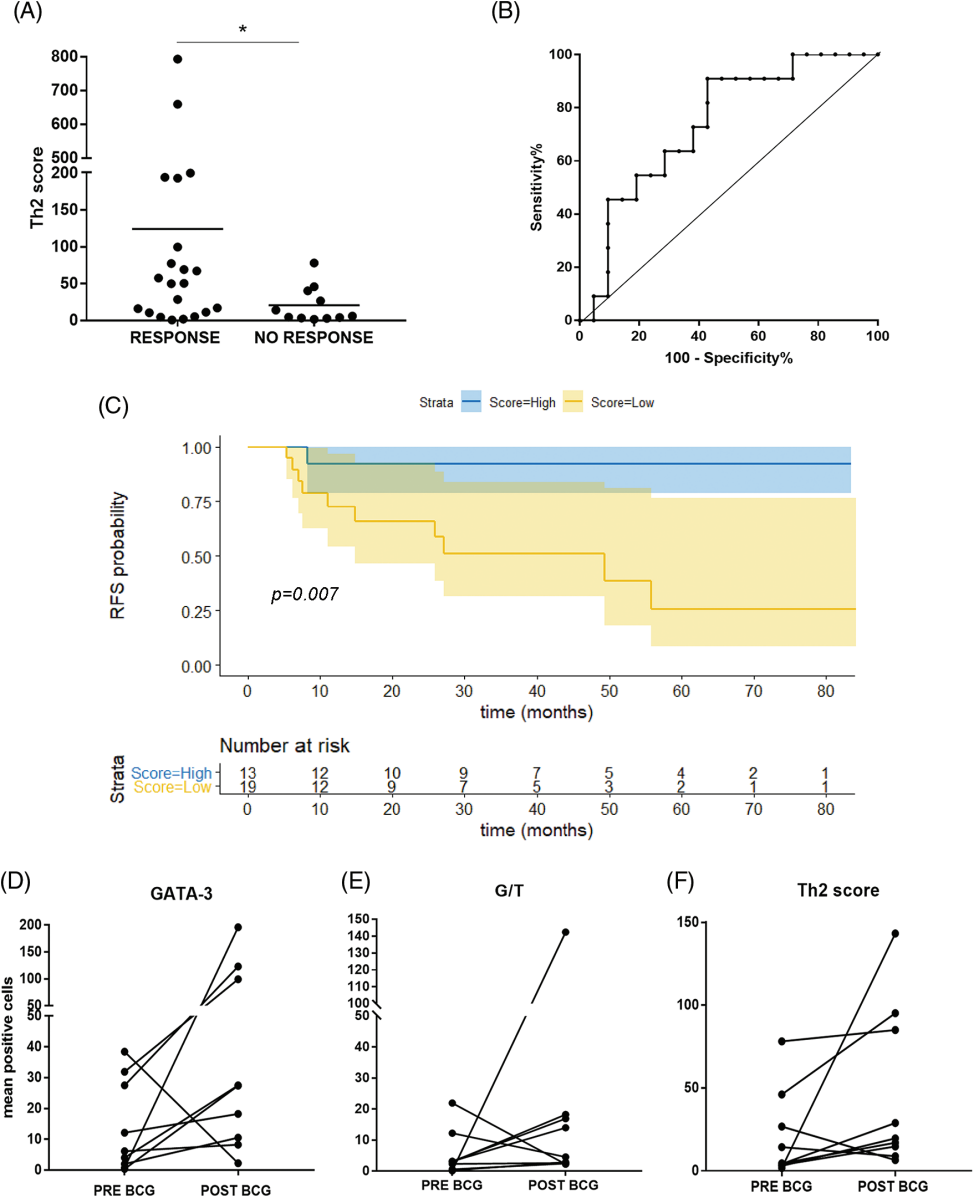
Th2-score in the TME can predict response to BCG in NMIBC patients. (A) Th2-score was higher in BCG-Responder than in BCG-NR non-responder patients (*p = 0.027, Mann–Whitney test); (B) ROC curve for the Th2-score allows discrimination of BCG-R patients (≥48.1) with 91% sensitivity and 57% specificity. (C) Kaplan Meier curve of recurrence-free survival (RFS) in months. Patients were stratified by Th2-score as low or high, based on the ROC curve cut-off (<48.1 = low count; ≥48.1 = high count) (*p* = 0.007, log-rank test (R); (D–F) Comparison of GATA-3 mean counts, G/T, and Th2-score in pre- and post-BCG biopsies (relapses), respectively.

When we combined the G/T ratio (TIL polarization) and the product of Eo counts and Eodgn, the BCG response was significantly associated with a higher Th2-score (*p* = 0.027) (Fig. 2A).

The AUC of the pre-BCG Th2-score was 0.74 (95% CI 0.56–0.91; *p* = 0.028). Since the predictive score was intended to identify true R and thus offer BCG treatment with high chances of success, a cutoff value for the Th2-score was set at >48.1, allowing discrimination of R with 91% sensitivity; however, specificity was 57.1% (PPV = 0.526, NPV = 0.923) (Fig. 2B).

**FIGURE S2:**
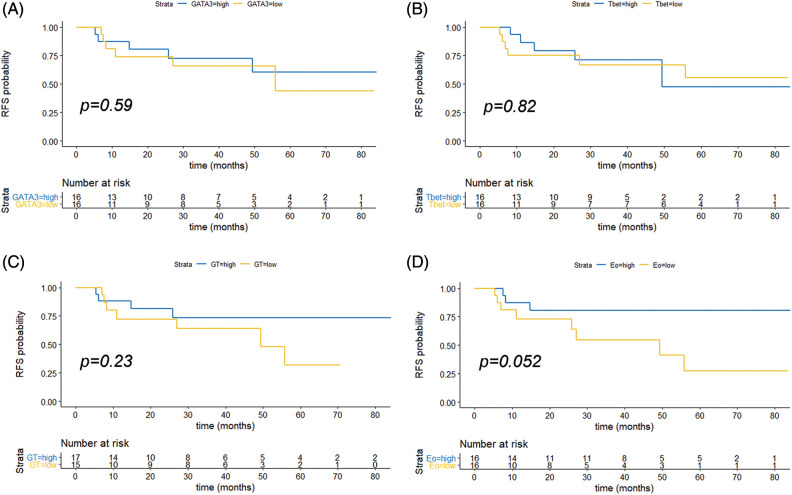
Kaplan Meyer curve of recurrence-free survival (RFS) in months. Patients were stratified by GATA-3 (A), T-Bet (B), G/T (C) and Eo counts (D) as low or high, based on the median values of the different variables analyzed (65.5 for age; 21.5 for GATA-3+; 2.9 for T-Bet+; 3.6 for G/T and 5.9 for EPX+). *p* values are indicated (log rank test, R).

For survival analyses, patients were divided into two groups (low *vs*. high counts) based on the median values of the different variables analyzed (65.5 for age; 21.5 for GATA-3+; 2.9 for T-Bet+; 3.6 for G/T and 5.9; EPX+) (Suppl. Table S3). No significant association was found for the other individual variables (GATA-3+, T-Bet+ cells, or G/T). Patients with high Eo counts tended to have a longer RFS than those with low Eo counts (*p* = 0.052) (Suppl. Fig. S2). Then, the patients were split into “high-Th2” and “low-Th2” score subgroups according to the cutoff value (48.1). Patients with a high Th2-score on pre-BCG biopsy had a significantly prolonged RFS compared with those with a low Th2-score (*p* = 0.007) (Fig. 2C).

Pre-BCG biopsies were compared to post-BCG biopsies in most NR cases (n = 9). The median time to recurrence was 8 months. Of note, in 8/9 tumors (88.9%), GATA-3+ TILs increased after recurrence (*p* = 0.055) (Fig. 2D). Accordingly, the G/T and Th2-score were also higher in 7/9 post-BCG biopsies (77.8%) as compared to pre-BCG tumors; however, these differences were not statistically significant (Figs. 2E and 2F, Suppl. Table S4).

### PD-1 and PD-L1 expression in NMIBC biopsies and response to BCG

**Figure 3:**
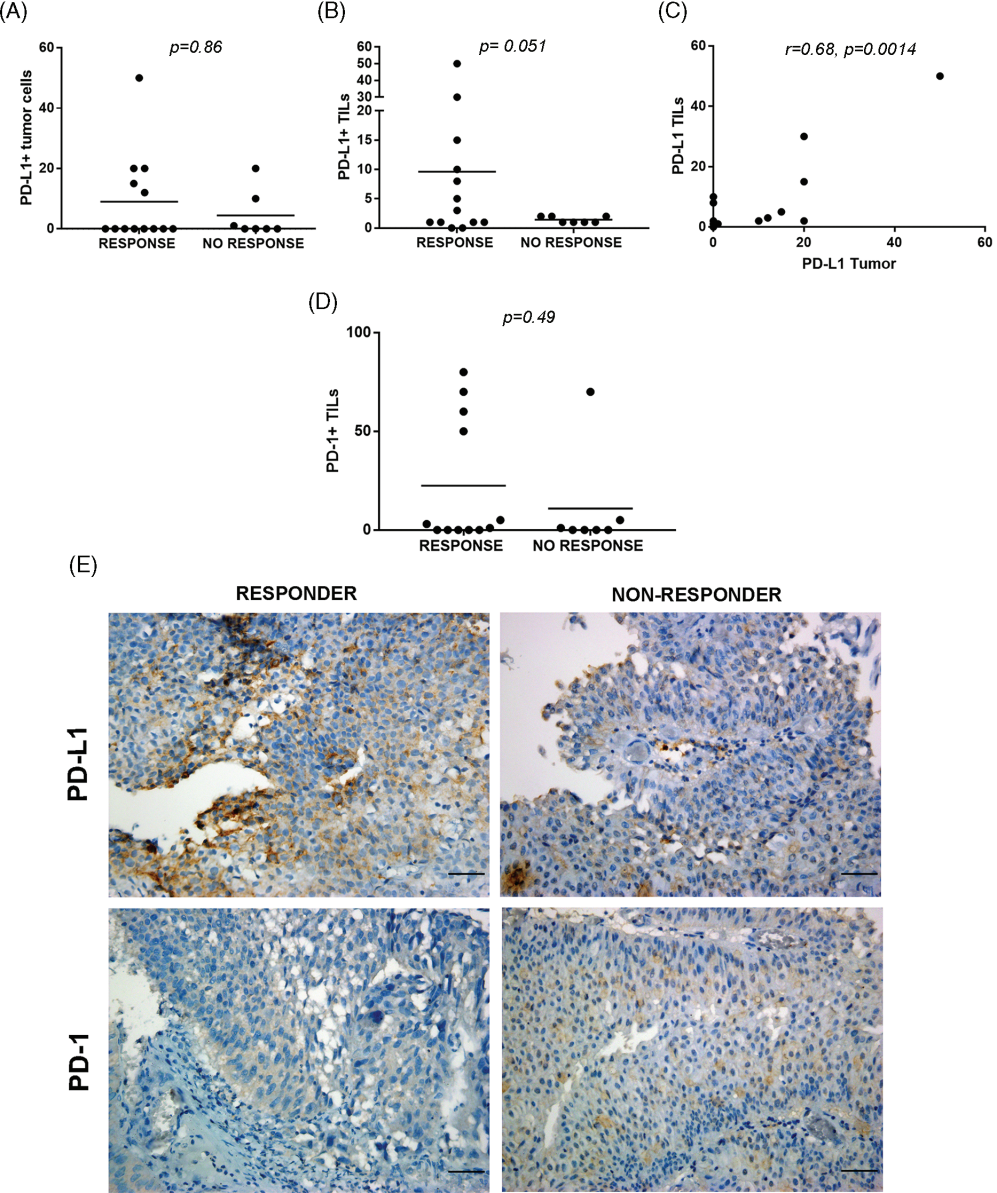
PD-L1/PD-1 expression in pre-BCG TILs of NMIBC biopsies. Quantitation of PD-L1 in tumor cells (A) and TILs (B) performed in BCG R or NR. (C) A positive correlation was found between PD-L1+ tumor cells and PD-L1+ TILs (C, r = 0.68, *p* = 0.0014, Spearman correlation test). (D) Invasive urothelial carcinoma showing PD-L1+ expression at the plasma membranes (left) and stromal TILs expressing PD-L1 in proximity to tumor cells (right). (E) Representative pictures of immunohistochemical staining of PD-L1 and PD-1 of pre-BCG tumor biopsies of BCG-R and BCG-NR patients. A, B, and D were compared with the Mann–Whitney test. Original magnification 400×. Scale bar = 50 μm.

Due to limited tumor availability, PD-L1/PD-1 staining was evaluated in 20 and 19 pre-BCG biopsies, respectively. PD-L1+ tumor cells were observed only in 8/20 tumors (40%), but they were detected in TILs in 15/20 biopsies (75%). No difference was observed in PD-L1+ tumor cells between BCG-R and BCG-NR patients (Fig. 3A), but a higher proportion of biopsies from R expressed PD-L1 in >5% of TILs than in NR (*p* = 0.051) (Fig. 3B).

PD-L1+ tumor cells were correlated with PD-L1+ TILs in all pre-BCG biopsies (r = 0.68, *p* = 0.0014), but these values were not associated with BCG response (Fig. 3C, Suppl. Table S5). More than 50% PD-1+ TILs were found in 4/12 biopsies from BCG-R patients, but only in 1/7 of BCG-NR, although this difference was not statistically significant (Fig. 3D). Representative images of PD-L1 and PD-1 expression in BCG-R and BCG-NR patients are shown in Fig. 3E. PD-1+ or PD-L1+ TILs were not correlated with GATA-3 or T-Bet expression or with the Th2-score (*p* > 0.05), regardless of the BCG response.

In post-BCG samples of BCG-NR patients, tumor cells and TILs showed no PD-L1 expression (0%–3%), and PD-1+ TILs were <1% (Suppl. Table S6). These results suggest that the PD-1/PD-L1 pathway was not involved in BCG failure.

## Discussion

There is a real need to identify patients with NMIBC that will not benefit from BCG immunotherapy, in order to offer them alternative treatments or clinical trials. *In vitro* and *in vivo* evidence supports that BCG acts as a potent Th1 inducer in the TME and is presumably responsible for long-lasting disease control [9,22]. However, the nature of the tumor antigens that are targets of the cytotoxic immune response or whether the anti-BCG immune response is involved in tumor elimination is still unknown. Additionally, experimental evidence has shown a direct effect of BCG in killing bladder cancer cells [23].

In the search for a possible biomarker of response to BCG, we evaluated lymphocyte polarization in the TME, along with the quantitation of eosinophils (associated with a Th2 immune microenvironment), at the maximum focus of infiltration, by IHC in pre-BCG biopsies of patients who had high-risk NMIBC, including HG pTa, LG/HG pT, and C*is* tumors, and received adequate BCG. Our goal was to define a Th2-score associated with BCG response and set a cutoff to predict true BCG-R patients with >90% sensitivity. Patients with higher Th2-scores showed significantly higher RFS after BCG treatment. Nunez-Nateras et al. [14] reported that a Th2-score obtained after quantitation of the Th2/Th1 lymphocyte ratio plus eosinophilic infiltration of pre-BCG biopsies was associated with BCG response in 38 NMIBC patients with C*is*. The authors hypothesized that a pre-existing Th2 TME would be susceptible to Th1 polarization, and therefore, Th2 status would correlate with the response to BCG treatment. However, in that study, patients received only one course of induction ×6, which is inadequate today. Of note, BCG response was assessed only after a short follow-up (6–8 weeks after BCG induction). Another study confirmed Nunez-Nateras’s findings [24]. However, the small number of patients included (19 BCG-R and 4 BCG-NR) may limit the conclusions, and the fact that only 25% of the patients had C*is* makes comparison difficult. Another report on 22 HG pT1 patients (none of them had C*is* tumors) showed that BCG-R patients had a significantly lower absolute number of peritumoral T-Bet+ cells and a higher G/T ratio [17]. However, the BCG strain and the treatment scheme were not specified. Furthermore, TILs G/T ratio adjusted for the systemic inflammation (neutrophil-to-lymphocyte ratio) showed a significantly higher association with the BCG response, a prolonged RFS in patients with a lower T-Bet+/Lymphocyte ratio, and higher GTR/NLR. In a later study [25], which included only 19 BCG-R and 4 BCG-NR patients the authors analyzed differences in the expression of GATA-3+ and T-Bet+ TILs (only evaluated in the lamina propria) between BCG-R *vs*. BCG-NR and found a clear tendency toward increased GATA-3+ T cell counts and GATA-3/T-Bet ratio.

Taken together, our results provide strong evidence that, after surgery, BCG can eradicate NMIBC cells only when the TME converts from Th2 to Th1.

Interestingly, we observed an increase in Th2 TILs in post-BCG biopsies from relapsed patients, probably reflecting the failure of BCG to induce a pro-inflammatory status. Presumably, the residual tumor cells that established strong immunosuppression sustained Th2 polarization.

Emerging data highlight that eosinophils infiltrate multiple tumors and display pleiotropic and even opposing roles (pro- *vs*. anti-tumorigenic activities) [26]. Furthermore, crosstalk between activated eosinophils and T cells increases CD8+ T cell migration to TME [27]. In addition, in patients treated with immune checkpoint inhibitors, increased eosinophilia is associated with responsiveness to therapy [27]. Thus, a higher pre-BCG Eo count in R patients could represent a Th2 polarized TME that can be shifted to Th1 by BCG administration, and eosinophils may contribute to Th1 cell recruitment. However, it was reported that blood eosinophils in patients with NMIBC could predict disease recurrence during BCG immunotherapy [28]. More data are needed to clarify this point.

Immune checkpoint upregulation is associated with a lack of antitumor cytotoxicity [29]. The upregulation of PD-L1 allows tumor cells to escape immune surveillance; thus, PD-L1 expression in the pretreatment TME could be a mechanism of BCG resistance. In our study, PD-L1+ tumor cells in pre-BCG biopsies were not associated with BCG response, in accordance with a previous report [17]. In our exploratory analysis, however, a higher proportion of BCG-R patients had >5% PD-L1+TILs than those from BCG-NR patients. Our findings are in disagreement with another report that found elevated PD-L1 expression in CD8+ TILs in baseline biopsies of BCG-NR patients [30]. However, we used a different anti-PD-L1 antibody (Ventana clone SP263 instead of Spring Biosciences clone SP142 or DAKO clone 22C3) and analyzed fewer patients. We observed >50% PD-1+TILs in a higher proportion of BCG-R than of BCG-NR patients, although this difference was not statistically significant, probably due to the small number of cases analyzed. In the post-BCG biopsies of relapsed patients, no exhausted PD-1+ TILs were detected. A possible explanation is that prior to BCG, an antitumor immune response was generated in some patients, accounting for the detection of exhausted T cells. However, an induced Th2 TME provides a cytokine balance that precludes the cytotoxic activity of TILs. In addition, PD-L1+ cells in the TME could contribute to the further abrogation of the antitumor response. Although our results suggest that the PD-1/PD-L1 pathway was not involved in BCG failure, this finding must be confirmed in a larger cohort of patients. Treatment with BCG shifts this balance toward Th1 polarization in BCG-R, patients increasing the affluence of innate and adaptive immune cells. Th1 cytokine production (i.e., IFN-γ) may fuel the elimination of residual tumor cells by immune effectors. Instead, in pre-BCG tumors with Th1-profiled TILs, other immunosuppressive mechanisms, such as secretion of TGF-β, PGE2, IL-10, and IL-6, as well as accumulation of myeloid-derived suppressor cells, tumor-associated macrophages, and regulatory T cells, could contribute to creating a highly tolerogenic TME [31]. Due to the small number of tumors available, we were not able to evaluate these immune populations in pre-BCG biopsies.

The limitations of this study are primarily associated with its retrospective design since only FFPE tumor biopsies were available for IHC analyses. In addition, the small number of patients reduced its statistical power. Recently, in the search for a biomarker profile associated with NMIBC patients’ response to BCG, we have initiated a prospective research study to validate the Th2-score combined with the analysis of immune cells and cytokines from urine and blood samples, obtained before and during treatment. Our results may have the potential to translate into a better selection of NMIBC patients to receive BCG treatment.

## Data Availability

All data generated or analyzed during this study are included in this published article and its supplementary files.

## Supplementary Materials

**Table S1:** Pre-BCG biopsies raw data

Patient	Gender	Age	Pathological stage	Histological grade	GATA-3 (mean)	T-Bet (mean)	G/T	Eo count (mean)	Eo dgn (max)	Eo count *Eo dgn	Th2- score	BCG response	Time to relapse/follow-up (months)
1	F	56	pT1	HG	4,000	1,250	3,200	1,38	1	1,38	4,58	NR	56
2	M	71	pT1	HG	31,875	134,000	0,238	3,88	1	3,88	4,11	NR	51
3	M	56	pTa	HG	12,125	20,000	0,606	38,75	2	77,50	78,11	NR	8
4	M	73	pTa + Cis	LG	4,250	3,000	1,417	9,38	1	9,38	10,79	R	>36
5	M	77	pTa	HG	109,125	9,875	11,051	0,25	1	0,25	11,30	R	9
6	M	76	pT1	HG	0,500	2,875	0,174	28,50	1	28,50	28,67	R	>69
7	F	65	pT1	HG	30,875	0,500	61,750	7,50	1	7,50	69,25	R	>70
8	F	60	pT1	HG	194,000	132,625	1,463	96,50	2	193,00	194,46	R	>71
9	M	62	pT1	HG	24,000	0,125	192,000	1,13	1	1,13	193,13	R	>60
10	M	61	pT1	HG	5,500	3,000	1,833	28,00	2	56,00	57,83	R	>55
11	F	44	pT1	HG	0,875	5,875	0,149	0,63	1	0,63	0,77	R	>9
12	M	70	pT1 + Cis	HG	29,750	4,375	6,800	9,38	1	9,38	16,18	R	>98
13	M	56	pT1	HG	38,375	1,750	21,929	4,75	1	4,75	26,68	NR	6
14	M	67	pT1	HG	59,000	0,125	472,000	94,00	2	188,00	660,00	R	>15
15	F	52	pT1	HG	12,000	0,125	96,000	3,88	1	3,88	99,88	R	>84
16	M	66	pT1	HG	0,625	0,500	1,250	0,88	1	0,88	2,13	R	>9
17	M	61	pT1	HG	19,000	9,250	2,054	3,25	1	3,25	5,30	R	>35
18	F	57	pT1	HG	6,000	1,375	4,364	22,88	2	45,75	50,11	R	>14
19	M	52	Cis	HG	27,875	5,750	4,848	1,25	1	1,25	6,10	NR	26
20	M	70	pT1	HG	8,875	17,000	0,522	38,50	2	77,00	77,52	R	>37
21	F	53	pT1	LG	27,500	2,250	12,222	2,13	1	2,13	14,35	NR	6
22	F	70	pT1	LG	2,000	0,625	3,200	0,38	1	0,38	3,58	NR	27
23	F	61	pTa	LG	0,750	7,125	0,105	1,63	1	1,63	1,73	NR	11
24	M	68	pTa	LG	0,375	0,125	3,000	0,00	0	0,00	3,00	NR	7
25	F	62	pT1	HG	82,250	0,125	658,000	67,75	2	135,50	793,50	R	>120
26	M	68	pT1	HG	6,125	2,625	2,333	21,88	2	43,75	46,08	NR	7
27	M	72	T1	HG	13,000	0,125	104,000	48,13	2	96,25	200,25	R	>47
28	M	82	Cis	HG	48,000	13,875	3,459	6,88	2	13,75	17,21	R	>41
29	F	67	pTa	HG	85,875	5,375	15,977	12,25	2	24,50	40,48	NR	15
30	M	72	pT1	HG	101,125	1,500	67,417	4,88	0	0,00	67,42	R	>30
31	F	31	pT1	LG	67,875	18,000	3,771	1,00	1	1,00	4,77	R	>17
32	M	74	pT1	HG	79,875	8,375	9,537	41,13	1	41,13	50,66	R	>32

Note: F = female; M = male; Grade: HG = high grade; LG = Low grade; R = BCG responder; NR = BCG non-responder; Th2-score = G/T (Eo count*Eo dgn).

**Table S2:** Correlation between individual variables in all pre-BCG biopsies

	Spearman r	*p* value
GATA-3 *vs.* T-BET	0.27	0.14
GATA-3 *vs.* Eo count	0.3	0.09
GATA-3 *vs.* Eodgn	0.18	0.31
T-BET *vs.* Eo count	0.05	0.77
T-BET *vs.* Eodgn	0.13	0.47
Eo count *vs.* Eodgn	0.71	5.6 × 10^–6 ****^

Note: **** highly significant.

**Table S3:** Data for Kaplan Meier analysis

Patient	Event R = 0; NR = 1	Event date	Diagnosis date	Score Th2	GATA-3	T-Bet	G/T	Eo count
1	1	06/01/15	10/2/10	Low	low	low	low	low
2	1	05/03/14	3/19/10	Low	high	high	low	low
3	1	05/11/11	9/1/10	High	low	high	low	high
4	0	04/15/14	4/29/11	Low	low	high	high	high
5	0	01/18/13	4/18/12	Low	high	high	high	low
6	0	02/22/18	5/22/12	Low	low	low	low	high
7	0	03/01/18	5/23/12	High	high	low	high	high
8	0	05/03/18	6/8/12	High	high	high	low	high
9	0	01/25/18	4/10/13	High	high	low	high	low
10	0	03/01/20	5/17/16	High	low	high	low	high
11	0	04/12/14	7/12/13	Low	low	high	low	low
12	0	03/01/21	11/26/13	Low	high	high	high	high
13	1	07/01/14	12/26/13	Low	high	low	high	low
14	0	04/28/15	1/24/14	High	high	low	high	high
15	0	03/01/21	3/12/14	High	low	low	high	low
16	0	07/24/15	10/24/14	Low	low	low	low	low
17	0	04/19/18	5/5/15	Low	low	high	low	low
18	0	07/25/16	6/23/15	High	low	low	high	high
19	1	04/01/09	2/1/07	Low	high	high	high	low
20	0	12/12/17	11/25/14	High	low	high	low	high
21	1	03/05/18	9/22/17	Low	high	low	hifg	low
22	1	09/14/18	6/10/16	Low	low	low	low	low
23	1	02/03/16	3/3/15	Low	low	high	low	low
24	1	02/21/17	7/22/16	Low	low	low	low	low
25	0	03/11/16	12/3/10	High	high	low	high	high
26	1	11/25/16	4/8/16	Low	low	low	low	high
27	0	08/03/18	9/9/14	High	low	low	high	high
28	0	08/01/20	3/28/17	Low	high	high	low	high
29	1	05/08/19	2/10/18	Low	high	high	high	high
30	0	03/01/21	9/19/18	High	high	low	high	low
31	0	03/01/21	10/11/19	Low	high	high	high	low
32	0	03/01/21	7/20/18	High	high	high	high	high

**Table S4:** Post BCG biopsies (relapses)–Raw data

Patient	GATA-3 (mean)	T-Bet (mean)	G/T	Eo count (mean)	Eo dgn (max)	Eo count *Eo dgn	Th2-score
3	18,25	7,375	2,47	41,25	2	82,5	84,97
13	2,25	1	2,25	4,25	1	4,25	6,50
22	10,5	0,75	14,00	0,75	1	0,75	14,75
23	195,875	1,375	142,45	0,75	1	0,75	143,20
24	27,375	1,5	18,25	1,375	1	1,375	19,63
26	8,25	3,125	2,64	46,25	2	92,5	95,14
1	27,5	1,625	16,92	0	0	0	16,92
2	99,25	34,375	2,89	13	2	26	28,89
21	122,875	26,75	4,59	4,25	1	4,25	8,84

**Table S5:** PD-1/PD-L1 in pre-BCG biopsies

Patient	PDL1 + Tumor cells (%)	PDL1+ TILs (%)	PD-L1 combined positivity score	PD-1+ TILs (%)	BCG response R = responder NR=non-responder
1	0	1	1	0	NR
3	0	1	1	0	NR
4	50	50	100	70	R
6	0	8	8	0	R
7	0	0	0	nd	R
8	15	5	20	5	R
9	0	0	0	3	R
10	20	30	50	0	R
12	12	3	15	1	R
13	10	2	12	0	NR
14	20	15	35	0	R
15	0	1	1	60	R
18	0	1	1	50	R
20	0	1	1	0	R
22	0	1	1	0	NR
23	1	<1	1	5	NR
24	20	2	22	70	NR
25	0	1	1	80	R
26	0	2	2	1	NR
27	0	10	10	0	R

**Table S6:** PD-1/PD-L1 in post-BCG biopsies

Patient	PDL1 + Tumor cells (%)	PDL1+ TILs (%)	PD-L1 combined positivity score	PD-1+ TILs (%)	Th2-score
1	0	3	3	1	16.92
2	0	0	0	<1%	28.89
3	nd	nd	nd	nd	84.97
13	nd	nd	nd	nd	6.50
21	0	0	0	0	8.84
22	0	0	0	<1%	14.75
23	0	0	0	<1%	143.20
24	0	0	0	<1%	19.63
26	0	0	0	<1%	95.14
